# Supplementary material for: Interplay of kinetochores and catalysts drives rapid assembly of the mitotic checkpoint complex
Source: Nat Commun. 2025 May 24;16:4823. doi: 10.1038/s41467-025-59970-1 (PMC12102207; doi:10.1038/s41467-025-59970-1)
Supplement: Supplementary file 1 — Supplementary Information [file 41467_2025_59970_MOESM1_ESM.pdf]

Table 1 ***Concentration of species used in MCC assembly assays***

Panel	Curve	MCC (CDC20)*	MPS1	M1:M2	B1:B3	Catalyst-(P)?	^{R}KT (wt or mutant)	^{R}KT-(P) (wt or mutant)	Rapamicyn	ATP
4B	Blue	500 nM	50 nM	100 nM	100 nM	Yes	/	20 nM	200 nM	2 mM
	Red	500 nM	50 nM	100 nM	100 nM	Yes	/	/	200 nM	2 mM
4C	Light brown	100 nM	50 nM	100 nM	100 nM	Yes	/	/	200 nM	2 mM
	Light blue	100 nM	50 nM	40 nM	40 nM	Yes	/	/	200 nM	2 mM
	Green	100 nM	50 nM	20 nM	40 nM	Yes	/	/	200 nM	2 mM
	Violet	100 nM	50 nM	20 nM	20 nM	Yes	/	/	200 nM	2 mM
	Teal	100 nM	50 nM	10 nM	10 nM	Yes	/	/	200 nM	2 mM
4D	Blue	100 nM	50 nM	20 nM	40 nM	No	20 nM	/	200 nM	2 mM
	Green	100 nM	50 nM	20 nM	40 nM	No	20 nM	/	/	2 mM
	Grey	100 nM	50 nM	20 nM	40 nM	No	/	/	200 nM	2 mM
4F	Blue	100 nM	50 nM	20 nM	40 nM	No	20 nM	/	200 nM	2 mM
	Green	100 nM	50 nM	20 nM	40 nM	No	20 nM	/	200 nM	2 mM
	Grey	100 nM	50 nM	20 nM	40 nM	No	/	/	200 nM	2 mM
	Violet	100 nM	50 nM	20 nM	40 nM	No	20 nM	/	200 nM	2 mM
5C	Blue	100 nM	50 nM	20 nM	40 nM	No	20 nM	/	200 nM	2 mM
	Pea	100 nM	50 nM	20 nM	40 nM	No	20 nM	/	200 nM	2 mM
	Brown	100 nM	50 nM	20 nM	40 nM	No	20 nM	/	200 nM	2 mM
5D	Blue	100 nM	50 nM	20 nM	40 nM	No	20 nM	/	200 nM	2 mM
	Orange	100 nM	50 nM	20 nM	40 nM	No	20 nM	/	200 nM	2 mM
	Purple	100 nM	50 nM	20 nM	40 nM	No	20 nM	/	200 nM	2 mM
	Grey	100 nM	50 nM	20 nM	40 nM	No	/	/	200 nM	2 mM
6D	Blue	100 nM	50 nM	20 nM	40 nM	No	20 nM	/	200 nM	2 mM
	Purple	100 nM	50 nM	20 nM	40 nM	No	20 nM	/	200 nM	2 mM
	Light brown	100 nM	50 nM	20 nM	40 nM	No	/	/	200 nM	2 mM
	Pink	100 nM	50 nM	20 nM	40 nM	No	/	/	200 nM	2 mM
6E	Blue	100 nM	50 nM	20 nM	40 nM	No	20 nM	/	200 nM	2 mM
	Brown	100 nM	50 nM	20 nM	40 nM	No	20 nM	/	200 nM	2 mM
	Light brown	100 nM	50 nM	20 nM	40 nM	No	/	/	200 nM	2 mM
	Teal	100 nM	50 nM	20 nM	40 nM	No	/	/	200 nM	2 mM
6F	Blue	100 nM	50 nM	20 nM	40 nM	No	20 nM	/	200 nM	2 mM
	Light blue	100 nM	50 nM	20 nM	40 nM	No	20 nM	/	200 nM	2 mM
	Light brown	100 nM	50 nM	20 nM	40 nM	No	/	/	200 nM	2 mM
	Dark pink	100 nM	50 nM	20 nM	40 nM	No	/	/	200 nM	2 mM
7A	Blue	100 nM	50 nM	20 nM	40 nM	No	20 nM	/	200 nM	2 mM
	Grey	100 nM	/	20 nM	40 nM	No	20 nM	/	200 nM	2 mM
	Brown	100 nM	50 nM	20 nM	40 nM	No	20 nM	/	200 nM	2 mM
7C	Blue	100 nM	50 nM	20 nM	40 nM	No	20 nM	/	200 nM	2 mM
	Green	100 nM	50 nM	20 nM	40 nM	No	20 nM	/	200 nM	2 mM
	Purple	100 nM	50 nM	20 nM	40 nM	No	20 nM	/	200 nM	2 mM
	Yellow	100 nM	50 nM	20 nM	40 nM	No	20 nM	/	200 nM	2 mM
	Grey	100 nM	/	20 nM	40 nM	No	20 nM	/	200 nM	2 mM
S1F	Blue	500 nM	50 nM	100 nM	100 nM	Yes	/	20 nM	200 nM	2 mM
	Grey	500 nM	50 nM	100 nM	100 nM	Yes	/	20 nM	200 nM	2 mM
	Black	500 nM	50 nM	100 nM	100 nM	Yes	/	/	200 nM	2 mM
S1G	Blue	100 nM	50 nM	100 nM	100 nM	No	20 nM	/	200 nM	2 mM
	Orange	100 nM	50 nM	100 nM	100 nM	No	20 nM	/	200 nM	/
	Grey	100 nM	50 nM	100 nM	100 nM	No	/	/	200 nM	2 mM
S1H	Purple	100 nM	50 nM	100 nM	100 nM	Yes	/	20 nM	200 nM	2 mM

	Green	100 nM	50 nM	100 nM	100 nM	Yes	20 nM	/	200 nM	2 mM
	Light blue	100 nM	50 nM	100 nM	100 nM	No	/	20 nM	200 nM	2 mM
	Blue	100 nM	50 nM	100 nM	100 nM	No	20 nM	/	200 nM	2 mM
	Black	100 nM	50 nM	100 nM	100 nM	Yes	/	/	200 nM	2 mM
	Grey	100 nM	50 nM	100 nM	100 nM	No	/	/	200 nM	2 mM
S2A	Dark red	100 nM	50 nM	20 nM	40 nM	No	20 nM	/	/	2 mM
	Grey	100 nM	50 nM	20 nM	40 nM	No	20 nM	/	/	2 mM
	Pink	100 nM	50 nM	20 nM	40 nM	No	20 nM	/	/	2 mM
S2B	Dark red	100 nM	50 nM	20 nM	40 nM	No	20 nM	/	/	2 mM
	Green	100 nM	50 nM	20 nM	40 nM	No	/	/	/	2 mM
	Black	100 nM	50 nM	20 nM	40 nM	No	20 nM	/	/	2 mM
	Light brown	100 nM	50 nM	20 nM	40 nM	No	20 nM	/	/	2 mM
S2C-G	See relevant parameters in Figure									
S3C**	Blue	100 nM	50 nM	20 nM	40 nM	No	20 nM	/	200 nM	2 mM
	Purple	100 nM	50 nM	20 nM	40 nM	No	20 nM	/	200 nM	2 mM
	Grey	100 nM	50 nM	20 nM	40 nM	No	/	/	200 nM	2 mM
	Green	100 nM	50 nM	20 nM	40 nM	No	20 nM	/	/	2 mM
	Pink	100 nM	50 nM	20 nM	40 nM	No	20 nM	/	/	2 mM
	Light blue	100 nM	50 nM	20 nM	40 nM	No	/	/	/	2 mM
S4E	Dark blue	100 nM	50 nM	20 nM	40 nM	No	20 nM	/	200 nM	2 mM
	Purple	100 nM	50 nM	20 nM	40 nM	No	20 nM	/	200 nM	2 mM
	Light blue	100 nM	50 nM	20 nM	40 nM	No	/	/	200 nM	2 mM
	Green	100 nM	50 nM	20 nM	40 nM	No	/	/	200 nM	2 mM
S4F	Dark blue	100 nM	50 nM	20 nM	40 nM	No	20 nM	/	200 nM	2 mM
	Grey	100 nM	50 nM	20 nM	40 nM	No	20 nM	/	200 nM	2 mM
	Light blue	100 nM	50 nM	20 nM	40 nM	No	/	/	200 nM	2 mM
	Salmon	100 nM	50 nM	20 nM	40 nM	No	/	/	200 nM	2 mM
S4G	Dark blue	100 nM	50 nM	20 nM	40 nM	No	20 nM	/	200 nM	2 mM
	Orange	100 nM	50 nM	20 nM	40 nM	No	20 nM	/	200 nM	2 mM
	Light blue	100 nM	50 nM	20 nM	40 nM	No	/	/	200 nM	2 mM
	Pink	100 nM	50 nM	20 nM	40 nM	No	/	/	200 nM	2 mM

*MAD2 and BUBR1 concentration were held at the fixed concentrations of 100 nM each in all experiments
**CCAN added at a concentration of 10 nM as indicated in Figure

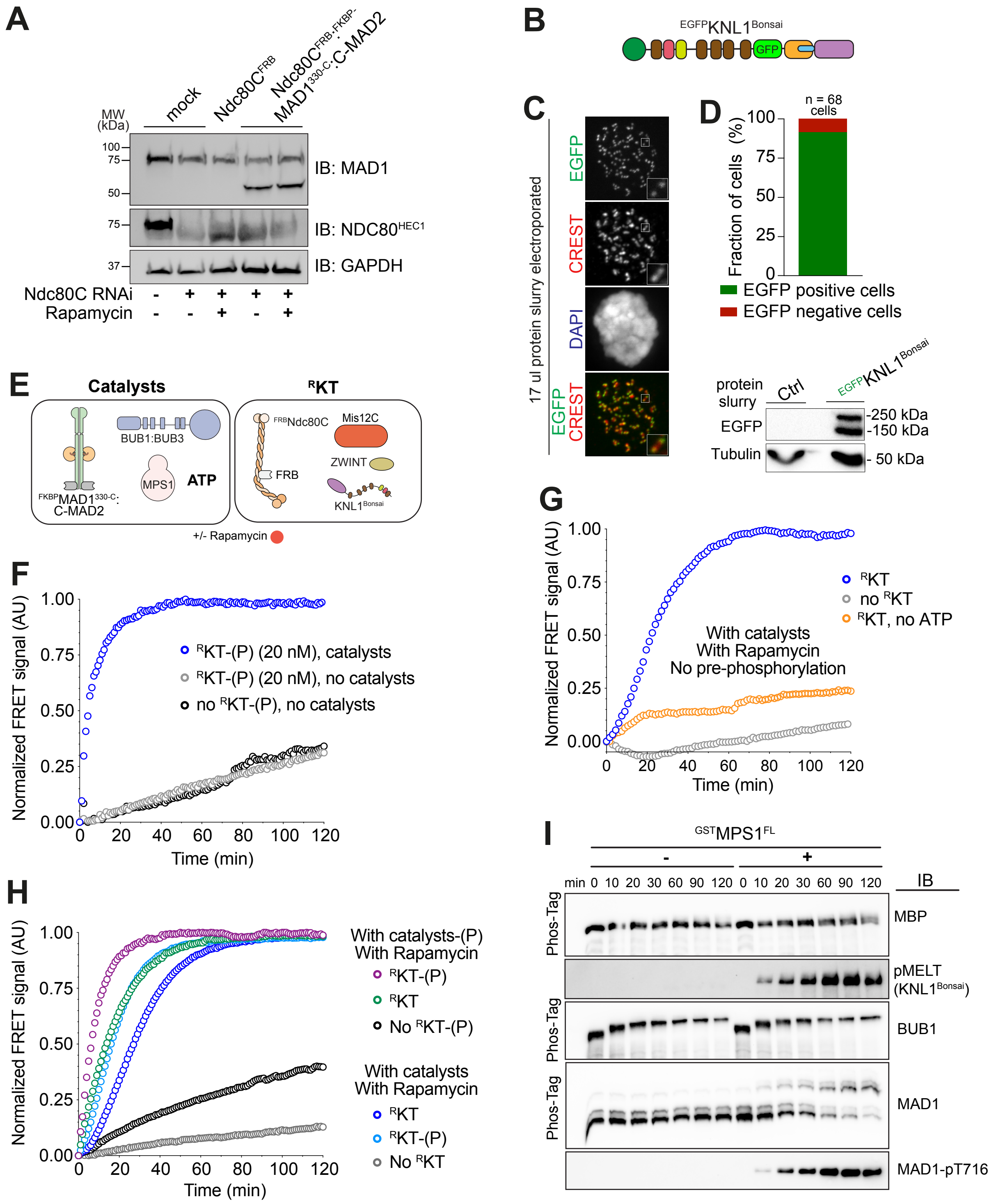
A
mock
Ndc80C^FRB
Ndc80C^FRB:FKBP-MAD1^330-C:C-MAD2
MW (kDa)
100
75
50
37
IB: MAD1
IB: NDC80^HEC1
IB: GAPDH
Ndc80C RNAi
Rapamycin
B
EGFPKNL1Bonsai
GFP
C
17 ul protein slurry electroporated
EGFP
CREST
DAPI
EGFP CREST
D
n = 68 cells
Fraction of cells (%)
EGFP positive cells
EGFP negative cells
protein slurry
Ctrl
EGFPKNL1Bonsai
EGFP
Tubulin
-250 kDa
-150 kDa
- 50 kDa
E
Catalysts
RKT
BUB1:BUB3
MPS1
ATP
FKBPMAD1330-C:C-MAD2
FRBNdc80C
Mis12C
ZWINT
FRB
KNL1Bonsai
+/- Rapamycin
F
Normalized FRET signal (AU)
Time (min)
RKT-(P) (20 nM), catalysts
RKT-(P) (20 nM), no catalysts
no RKT-(P), no catalysts
G
RKT
no RKT
RKT, no ATP
With catalysts
With Rapamycin
No pre-phosphorylation
H
With catalysts-(P)
With Rapamycin
RKT-(P)
RKT
No RKT-(P)
With catalysts
With Rapamycin
RKT
RKT-(P)
No RKT
I
GSTMPS1FL
min 0 10 20 30 60 90 120 0 10 20 30 60 90 120
IB
Phos-Tag
MBP
pMELT (KNL1Bonsai)
BUB1
MAD1
MAD1-pT716

Figure S1

Figure S1. ***Further characterization of*** ^{R}***KTs***

A) Western blots displaying controls for RNAi and electroporation experiments in Figure 2C-F.

B) Scheme of EGFP-labelled KNL1^{Bonsai} used in panel **C**.

C) Representative image of HeLa cells electroporated with ^{GFP}KNL1^{Bonsai} (at slurry concentration of 6 μM). DAPI and CREST stain DNA and inner kinetochores, respectively. Scale bar: 5 μM.

D) Quantification of electroporation efficiency with immunoblot from 50 μg cleared mitotic lysate from HeLa cells electroporated with ^{GFP}KNL1^{Bonsai}. Tubulin is the loading control. The two bands in the anti-GFP blot represent ^{EGFP}KNL1^{Bonsai} and unfused ^{EGFP}KNL1^{M5}.

E) Scheme of proteins used in assays in panels F-H.

F) FRET curves for MCC sensor in presence of pre-phosphorylated catalysts, or in conditions where catalysts (grey), or both catalysts and kinetochores (black), were omitted. Catalytic components or kinetochores were prephosphorylated with MPS1 as indicated. The blue curve was normalized to its own maximum. The grey and black curves were normalized to maximum of the blue curve.

G) FRET assay monitoring MCC assembly with (blue curve, already shown in Figure 4D) or without (grey curve; already shown in Figure 4D) reconstituted ^{R}KTs, and in absence of ATP (orange). MAD1:MAD2, BUB1:BUB3 and ^{R}KT were not pre-phosphorylated with MPS1.

H) FRET assay monitoring rate of MCC formation with MPS1-pre-phosphorylated catalysts (20 nM MAD1:MAD2 and 40 nM BUB1:BUB3; purple, green and black curves) or not pre-phosphorylated catalysts (blue, light blue, grey curves). ^{R}KT, pre- or not pre-phosphorylated, were added where indicated. Purple, green, light blue and blue curves were normalized to their own maximum. The black and grey curve were normalized to maximum of green curve. Panels reporting time-dependent changes in FRET signal are from single measurements and representative of at least three independent technical replicates.

I) Western blots of phosphorylation targets and phosphorylated residues. Phos-Tag SDS-PAGE emphasizes changes in electrophoretic mobility upon phosphorylation. The kinase reaction was performed under standard conditions monitoring MCC assembly in the plate reader (20 nM ^{R}KT in, 20 nM MAD1:MAD2, 40 nM BUB1:BUB3, 50 nM MPS1, 100 nM CDC20, 200 nM Rapamycin, 2 mM ATP, 10 mM $MgCl_2$), but in absence of fluorescent subunits (^{CFP}BUBR1 and MAD2^{TAMRA}). Experiment repeated in triplicate.

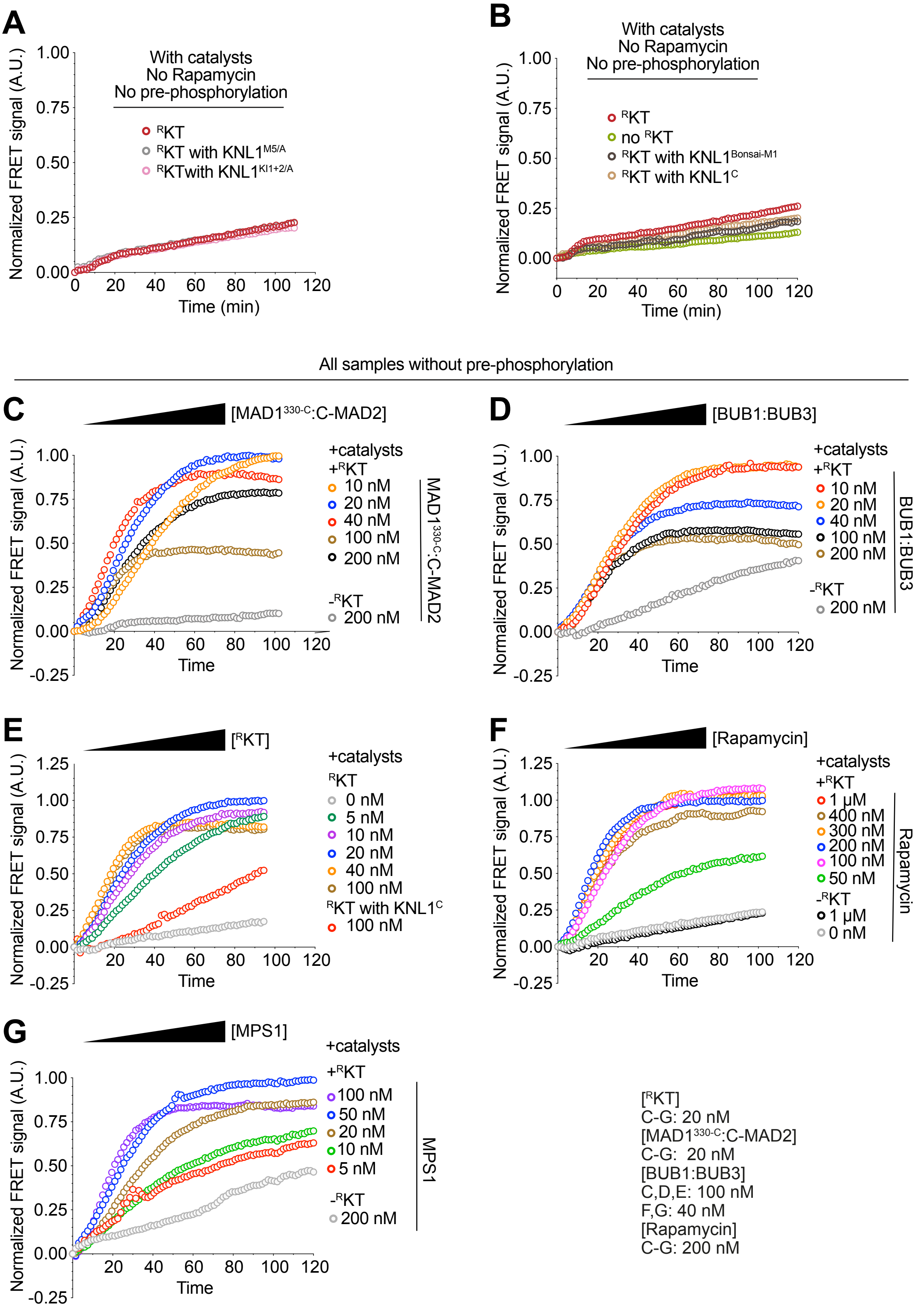

[RKT]
C-G: 20 nM
[MAD1330-C:C-MAD2]
C-G: 20 nM
[BUB1:BUB3]
C,D,E: 100 nM
F,G: 40 nM
[Rapamycin]
C-G: 200 nM

Figure S2

Figure S2. ***Rate of MCC assembly under various conditions***

A) Additional controls for experiments with ^{R}KT performed in Figure 5C. FRET assay comparing the MCC catalytic assembly with wild-type ^{R}KT (maroon), ^{R}KT with Knl1$^{KI1+2/A}$ (where KI1 and KI2 motifs of KNL1 are mutated, pink) and ^{R}KT with Knl1$^{M5\text{-}A}$ (where five MELT motifs of KNL1 are mutated, grey) without Rapamycin.

B) Additional controls for experiments with ^{R}KT performed in Figure 5D. FRET assay comparing the MCC catalytic assembly with wild-type ^{R}KT (maroon), ^{R}KT with Knl1$^{M1/A}$ (where only first MELT motif is present, dark grey), ^{R}KT with Knl1^{C} (where only C terminal of KNL1 is present light brown), and no ^{R}KT (light green) in the absence of Rapamycin.

C-**G**) Effect of titration of the indicated catalyst concentration, Rapamycin, and ^{R}KT on MCC assembly rate. Panels reporting time-dependent changes in FRET signal are from single measurements and representative of at least three independent technical replicates of the experiment.

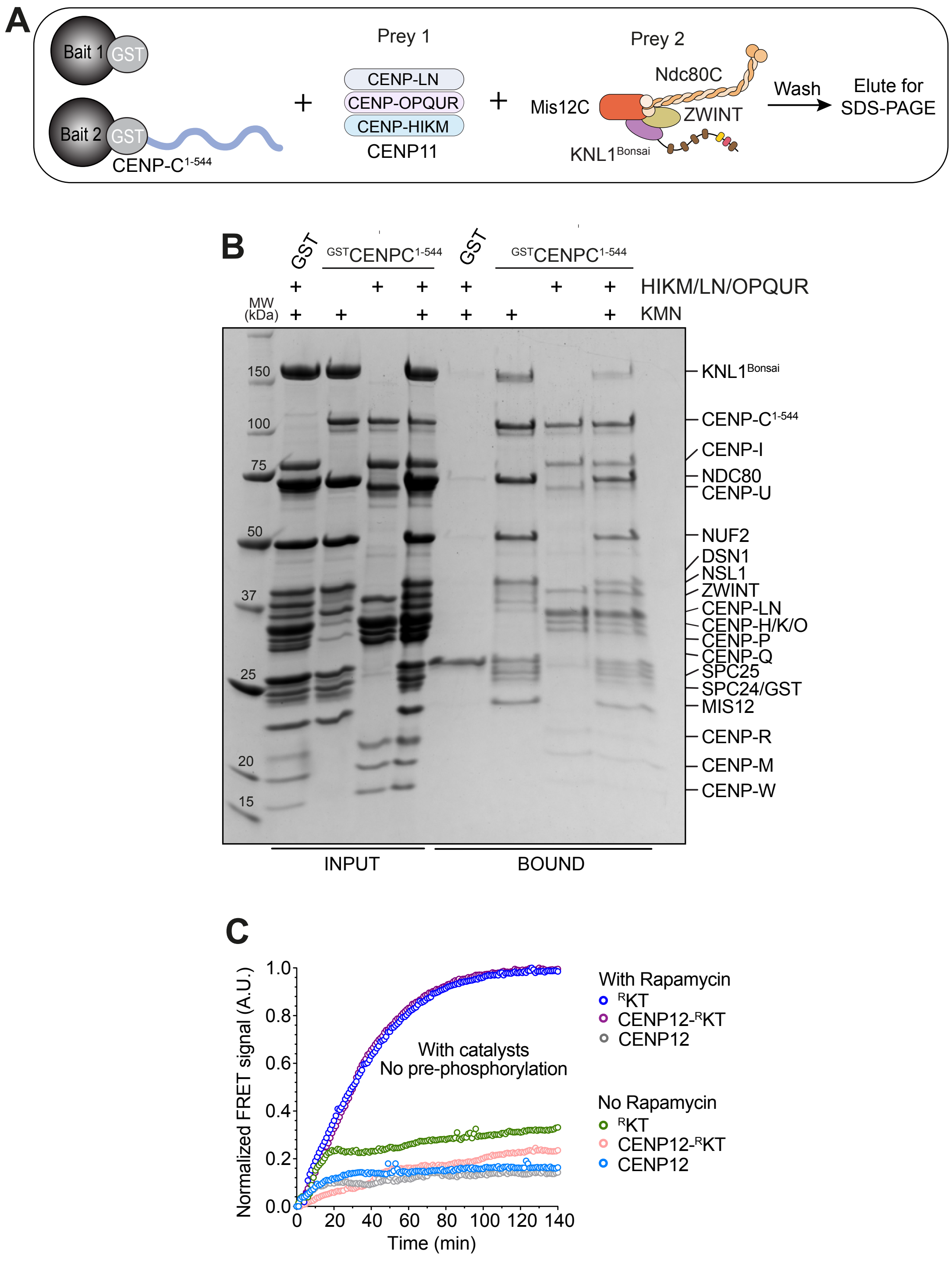
A
Bait 1
GST
Bait 2
GST
CENP-C1-544
Prey 1
CENP-LN
CENP-OPQUR
CENP-HIKM
CENP11
Prey 2
Ndc80C
Mis12C
ZWINT
KNL1Bonsai
Wash
Elute for SDS-PAGE
B
GST
GSTCENPC1-544
GST
GSTCENPC1-544
HIKM/LN/OPQUR
KMN
MW (kDa)
150
100
75
50
37
25
20
15
KNL1Bonsai
CENP-C1-544
CENP-I
NDC80
CENP-U
NUF2
DSN1
NSL1
ZWINT
CENP-LN
CENP-H/K/O
CENP-P
CENP-Q
SPC25
SPC24/GST
MIS12
CENP-R
CENP-M
CENP-W
INPUT
BOUND
C
Normalized FRET signal (A.U.)
Time (min)
With catalysts
No pre-phosphorylation
With Rapamycin
RKT
CENP12-RKT
CENP12
No Rapamycin
RKT
CENP12-RKT
CENP12

Figure S3

Figure S3. ***CCAN does not contribute to catalytic assembly of MCC***

A) Scheme of GST pulldown assays. GST or ^{GST}CENP-C were incubated with CENP12 on GSH beads as bait. ^{R}KT was added as the prey.

B) SDS-PAGE of GSH based pulldown with the indicated baits and preys. ^{GST}CENP-C$^{1\text{-}544}$ (3 μM) as a bait with CENP11 (CENP-HIKM/CENP-LN/CENP-OPQUR) (4 μM) and ^{R}KT (6 μM) as prey.

C) FRET assay monitoring the rate of MCC formation in the presence of the indicated CCAN species and in the presence of catalysts and ^{R}KT. The blue and purple curves were normalized to their maximum. Fluorescence values of the other curves were normalized to the maximum value of the blue curve. Panels reporting time-dependent changes in FRET signal are from single measurements and representative of at least three independent technical replicates of the experiment.

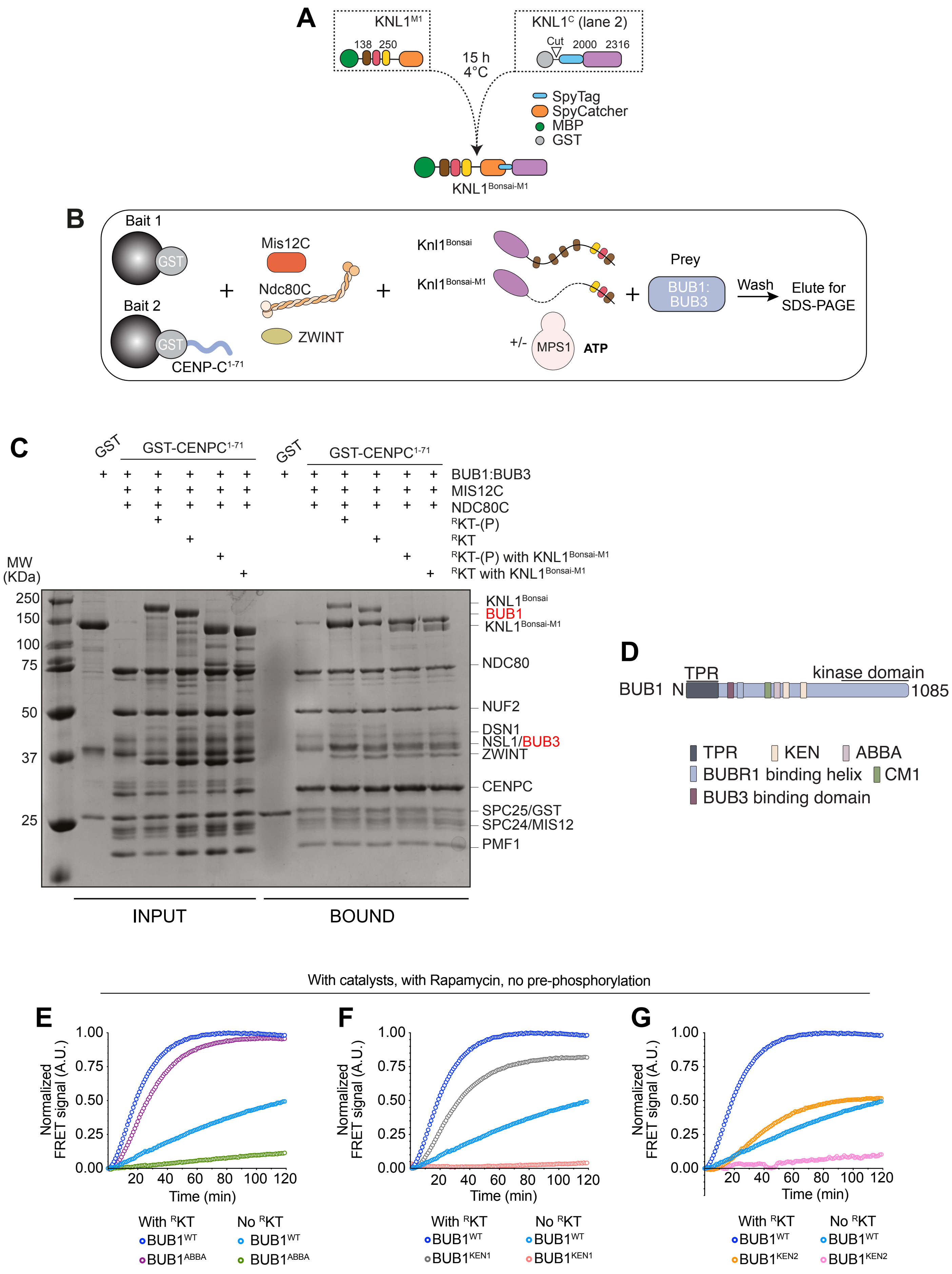
A
KNL1M1
138 250
KNL1C (lane 2)
Cut
2000 2316
15 h
4°C
SpyTag
SpyCatcher
MBP
GST
KNL1Bonsai-M1
B
Bait 1
GST
Bait 2
GST
CENP-C1-71
Mis12C
Ndc80C
ZWINT
Knl1Bonsai
Knl1Bonsai-M1
+/-
MPS1
ATP
Prey
BUB1:
BUB3
Wash
Elute for
SDS-PAGE
C
GST
GST-CENPC1-71
BUB1:BUB3
MIS12C
NDC80C
RKT-(P)
RKT
RKT-(P) with KNL1Bonsai-M1
RKT with KNL1Bonsai-M1
MW
(KDa)
250
150
100
75
50
37
25
KNL1Bonsai
BUB1
KNL1Bonsai-M1
NDC80
NUF2
DSN1
NSL1/BUB3
ZWINT
CENPC
SPC25/GST
SPC24/MIS12
PMF1
INPUT
BOUND
D
BUB1
N
TPR
kinase domain
1085
TPR
KEN
ABBA
BUBR1 binding helix
CM1
BUB3 binding domain
With catalysts, with Rapamycin, no pre-phosphorylation
E
F
G
Normalized
FRET signal (A.U.)
1.00
0.75
0.50
0.25
0.00
20 40 60 80 100 120
Time (min)
With RKT
No RKT
BUB1WT
BUB1ABBA
BUB1KEN1
BUB1KEN2

Figure S4

Figure S4. ***Additional experiments with KNL1 and BUB1 variants***

A) Scheme for the assembly of KNL1$^{\text{Bonsai-M1}}$.

B) Scheme for binding assays with ^{R}KT assembled with KNL1^{Bonsai} or KNL1$^{\text{Bonsai-M1}}$.

C) SDS-PAGE of BUB1 binding assays with ^{R}KT assembled with KNL1^{Bonsai} or KNL1$^{\text{Bonsai-M1}}$.

D) Scheme of BUB1 motifs and domains.

E) MCC FRET assay monitoring the assembly of MCC without pre-phosphorylation of the components by MPS1, in the presence of catalysts and Rapamycin with ^{R}KT (dark blue curve, also shown in panels **F** and **G**) and without ^{R}KT (light blue curve, also shown in panel **F** and **G**). The BUB1^{ABBA} mutant was tested with (purple curve) and without ^{R}KT (green curve). Experiment repeated in triplicate.

F) MCC FRET assay monitoring the assembly of MCC without pre-phosphorylation of the components by MPS1, in the presence of catalysts and Rapamycin with ^{R}KT (dark blue curve, also shown in panels **E** and **G**) and without ^{R}KT (light blue curve, also shown in panel **E** and **G**). The BUB1$^{\text{KEN1}}$ mutant was tested with (grey curve) and without ^{R}KT (salmon curve). Experiment repeated in triplicate.

G) MCC FRET assay monitoring the assembly of MCC without pre-phosphorylation of the components by MPS1, in the presence of catalysts and Rapamycin with ^{R}KT (dark blue curve, also shown in panels **E** and **F**) and without ^{R}KT (light blue curve, also shown in panel **E** and **F**). The BUB1$^{\text{KEN2}}$ mutant was tested with (orange curve) and without ^{R}KT (pink curve). Experiment repeated in triplicate.
